# Drug-dependence behaviour and outcome of medication-overuse headache after treatment

**DOI:** 10.1007/s10194-012-0492-z

**Published:** 2012-10-18

**Authors:** Ilenia Corbelli, Stefano Caproni, Paolo Eusebi, Paola Sarchielli

**Affiliations:** 1 Neurologic Clinic, Headache Centre, Santa Maria della Misericordia Hospital, University of Perugia, Sant’Andrea delle Fratte, 06158 San Sisto, Perugia Italy; 2 Epidemiology Department, Regional Health Authority of Umbria, Via Mario Angeloni 61, 06124 Perugia, Umbria Italy

**Keywords:** Medication-overuse headache, Detoxification, Symptomatic drugs overuse, Drug dependence, Leeds Dependence Questionnaire

## Abstract

This study aimed at determining the causes of failure of the different proposed strategies to ensure improvement of medication-overuse headache (MOH) patients, since they have not been investigated so far, especially with regard to aspects related to cognitive and behavioural aspects of symptomatic drugs overused by them. One hundred and twenty in-patients, 82 females (68.3 %), median age 49 (42–56) years, affected by MOH were admitted to the study and treated with abrupt discontinuation of the medication overused, a 6-day in-patient detoxification regimen and an immediate start of personalized prophylactic treatment, then followed for 1 year. Leeds Dependence Questionnaire (LDQ), among all the clinical variables, was administered at baseline and at 1-year follow-up visit to assess substance dependence. Of the 120 patients enrolled, 68 (56.7 %) were successfully detoxified (Responder-group), while 52 (43.3 %) were not (Non-Responder-group). At baseline, the mean LDQ total score was slightly higher in the Non-Responder group than in the Responder group (12.08 ± 2.14 vs. 11.94 ± 1.98). Although this difference was not significant at baseline (*p* > 0.05), the LDQ total score was significantly different (*p* < 0.001) at the 1-year follow-up visit between the responder group (7.8 ± 2.3) and the Non-Responder group (12.1 ± 2.1). Moreover, the pattern of the responses of the patients in the responder group differed from that of the Non-Responder-group in the items relating to the *compulsion to start*, *compulsion to continue*, *primacy of effect*, *constancy of state* and *cognitive set*. The results showed that patients of the Non-Responder group showed a drug dependence pattern similar to that previously described in addicts. Conversely, in patients who positively responded to the procedure, drug-abuse behaviour seemed to be a consequence of chronic headache, reflecting the need for daily analgesic use to cope with everyday life.

## Introduction

Medication-overuse headache (MOH) [1] and its treatment represent the major challenge for a physician in a specialty headache centre. Its prevalence is 1–1.4 % in the general population with a peak in women aged 40–50 years (with a prevalence of 5 % in this subgroup) [1–3], reaching up to 10 % of patients seen in headache clinics [4].

According to the International Classification of Headache Disorders (ICHD-II), MOH implies that headache is present on ≥15 days/month with a regular overuse for >3 months of one or more drugs that can be taken for acute/symptomatic treatment of headache (≥10 days/month for ergotamine, triptans, opioids, combination analgesic medications or combination of acute medications and ≥15 days/month for analgesics and nonsteroidal anti-inflammatory drugs—NSAIDs) [5–7].

The detoxification of the patient and the start of a prophylactic therapy is, nowadays, the standard of care all over the world [8, 9] though there is no established consensus concerning withdrawal and detoxification strategies in MOH [10].

Although the majority of patients report an improvement of headache shortly after withdrawal, long-term studies (involving follow-up periods of up to 6 years) indicate that between 24 and 43 % of them relapse (40 % during the first year after withdrawal) and develop MOH again despite an initially successful withdrawal therapy [8, 11–14].

Causes of failure of the different proposed regimens have only partially been investigated [4].

Predictors of relapse in MOH after withdrawal and detoxification are (1) type of primary headache (migraine patients had a lower relapse rate than patients with tension-type headache or a combination of both), (2) type of overused headache medication (combination of analgesics with codeine or barbiturates use had the higher rates of relapse), (3) female gender, (4) long duration of primary headache, (5) long duration of drug overuse and (6) psychiatric comorbidity [4, 8, 15–17]. In particular, the impaired control over the use of the substance and propensity to relapse long after withdrawal symptoms suggest that a behavioural disorder, such as substance dependence, play a major role in promoting and maintaining MOH [16].

In the literature, to date, there are clinical, pathophysiological and genetic data supporting a relationship between MOH and dependence-related behaviour [18]. A deep knowledge of these aspects can lead to clinical and therapeutic implications.

The Leeds Dependence Questionnaire (LDQ) is a self-completion, 10-item questionnaire, validated to be used in addiction and psychiatric settings for alcohol and opiate consumers to measure substance dependence severity [19, 20].

Some LDQ items were modified by Ferrari et al. [21] so that they could be applied to consumers of analgesics. These authors administered the LDQ to three groups of patients: chronic daily headache (CDH) and episodic headache groups and a drug addicts group. Interestingly, similar responses were found in the CDH and the drug addicted groups. The authors have postulated that CDH patients have a very strong need for analgesics, which is similar to the need found in drug addicts. More recently, the same group administered their modified version of the LDQ to episodic migraine patients, chronic migraine patients overusing acute medications and patients suffering from rheumatic disease [22].

The aim of the present study was to assess cognitive and behavioural aspects of symptomatic drug overuse in MOH patients. For this purpose, we administered the LDQ in MOH patients before and after a detoxification protocol followed by prophylactic treatment. We aimed to verify if a different pattern of drug overuse could be identified among patients which showed improvement as a consequence of undergoing the treatment protocol compared to those patients who did not show any improvement.

## Patients and methods

Our Institutional Review Board and local Ethical Committee approved the study and informed consent was obtained from the participants.

Consecutive new patients underwent a semi-structured, face-to-face interview in our Headache Centre (Neurologic Clinic, S.M. Misericordiae Hospital, Perugia, Italy). MOH diagnosis was made according to ICHD-II modified criteria [6] by two experienced clinicians of our Headache Centre based on a semi-structured clinical interview and a 3-month headache diary. Patients with serious concomitant disease, systemic pathologies, symptomatic headaches, a current or prior history of drug/alcohol or strong opioid [23] abuse, pregnant or breastfeeding and aged <18 or >65, were excluded. Included patients were treated with abrupt discontinuation of the medication overused, a 6-day in-patient detoxification regimen and an immediate start of personalized prophylactic treatment and then followed for 1 year.

The standard 6-day in-patient detoxification programme consisted of (1) verbal advice on abruptly withdrawing the overused medication, (2) 1,000 cc saline solution hydration i.v./daily and (3) metoclopramide 10 mg/daily, if needed [24]. During the in-patient detoxification phase, residual attacks were treated with a drug not involved in the drug abuse (an anti-inflammatory drug or a triptan).

Prophylactic drug classes used, chosen according to individual characteristics (compliance, comorbidities and tolerability), past experiences of preventive therapies or kind of their headache, included tricyclic antidepressants, beta-blockers, antiepileptics, selective serotonin reuptake inhibitors (SSRIs) and, in some cases, a combination of all of these. The treatment was maintained for a minimum period of 3 months. After the baseline visit, they underwent three intermediate visits (one every 3 months) to verify prophylactic treatment compliance and side effects, before the 1-year follow-up visit.

Patients were given a diary to record, on a daily basis, the occurrence, severity, duration of the headache episodes and the use of acute medications. Clinical variables assessed at baseline and at 1-year follow-up included number of days with headache per month, duration of headache, pain intensity measured with a 1–100 mm Visual Analogical Scale (0 = no pain, 100 mm = worst imaginable pain) and amount and type of abused drugs.

According to the Diagnostic and Statistical Manual of Mental Disorders (DSM)-IV criteria [25], psychiatric assessment prior to medication withdrawal included a semi-structured face-to-face interview for the evaluation of psychiatric comorbidity with Beck Anxiety Inventory and Beck Depression Inventory scales to verify the occurrence of anxiety and depression [26]. Moreover, the Modified Mini International Neuropsychiatric Interview (M.I.N.I.) [27] was administered.

The LDQ [19, 21] is composed of 10 items, which are scored with 4 digits: 0-1-2-3 (0 = never, 1 = sometimes, 2 = often, 3 = nearly always) measuring the severity of dependence upon substances, independent of the pharmacological properties or the quantity of substances overused. The operational definitions given to the 10 cognitive and behavioural markers of substance dependence by Raistrick et al. [19], representing the ICD-10 [20] and DSM-IV [25] criteria for substance dependence, are *pre*-*occupation*, *salience*, *compulsion to start*, *planning*, *maximize effect*, *narrowing of repertoire*, *compulsion to continue*, *primacy of effect*, *constancy of state* and *cognitive set* (items from 1 to 10, respectively). The LDQ total score increases with the degree of substance dependence, but no cut-off score indicating dependence has been identified. High LDQ scores are associated with cognitive preoccupation with substance use, a compulsion to use, continual use, planning and organizing future use, maximization of the subjective experience of substance use, a reduced repertoire of behaviour with the primacy of substance use and substance use as an existential coping strategy.

Other details on LDQ have been described previously [19, 28].

The LDQ was administered at baseline and at 1-year follow-up visit to assess substance dependence.

Based on clinical outcome at 1 year, two groups were identified:

- The Responder group (R-group)—Successful detoxification: patients with ≥50 % decrease in headache days/month from baseline, resolving medication overuse, within 2 months after detoxification and without relapse for the following year.
- The Non-Responder group (NR-group)—Unsuccessful detoxification: patients who returned to a pattern of medication overuse within 1 year and continued to complain of a chronic headache.

Specifically, we use the term “Responder” to identify those patients who responded to the treatment (i.e., an improvement of headache following detoxification regimen and prophylactic therapy, reverting chronic headache to an episodic pattern), as a consequence of treatment adherence, maintaining abstinence from MOH-inducing medications in the follow-up period, whereas the term “Non-Responder” refers to the patient group showing no benefits to the treatment both in terms of reduced headache days and medication overuse.

### Statistical analysis

Statistical analysis was performed using Statistics, Release 6.0. Continuous variables were tested for normality with Kolmogorov–Smirnov normality test. Comparisons between groups were made using *t* test for the majority of the parameters studied, which followed a normal distribution. Kruskal–Wallis test was used for age and age of headache onset variables, which were non-normally distributed. Categorical variables, shown in the Tables as percentages, are referred to in the specific column. Percentages between groups were compared by the Chi square test and Fisher’s exact test and *p* < 0.05 was chosen as the minimum level of statistical significance. A two-factor MANOVA with all LDQ items as dependent variables has been performed.

Bonferroni adjusted* t*-values and *p* values were reported for post hoc when pairwise-comparisons were performed in two-way analysis of variance.

## Results

A total of 129 patients were consecutively enrolled; 9 of them were excluded because they failed to appear at follow-up visits.

One-hundred and twenty patients completed the study, including 82 females (68.3 %), median age 49 (lower–upper quartiles = 42–56) years. There were no significant demographic differences between included and excluded patients. Before detoxification, 59 % of patients overused more than one type of acute medication.

Details of these 120 MOH patients at baseline are reported in Tables 1 and 2 display the subtype of acute treatments abused at baseline.

**Table 1:** Clinical details of MOH patients at baseline

	Total group	R-group	NR-group	Test statistics	*p*-value
Patients (*n*)	120	68 (56.7 %)	52 (43.3 %)	$\chi^{2} = 2.13$	0.1441
Males (*n*) Females (*n*)	38 (31.7 %) 82 (68.3 %)	24 (35.3 %) 44 (64.7 %)	14 (26.9 %) 38 (73.1 %)	$\chi^{2} = 0.95$	0.3286
Age (years)	49 (42–56)	50.5 (44–55)	50 (39–54)	$\chi^{2} = 1.22$	0.2690
Marital status					
Married	67 (55.8 %)	37 (54.4 %)	30 (57.7 %)	$\chi^{2} = 0.44$	0.9312
Unmarried	39 (32.5 %)	22 (32.4 %)	17 (32.7 %)		
Divorced	11 (9.2 %)	7 (10.3 %)	4 (7.7 %)		
Widowed	3 (2.5 %)	2 (2.9 %)	1 (1.9 %)		
Education					
Primary school	21 (17.5 %)	12 (17.6 %)	9 (17.3 %)	$\chi^{2} = 0.36$	0.9484
Secondary school	38 (31.7 %)	22 (32.4 %)	16 (30.8 %)		
High school	37 (30.8 %)	20 (29.4 %)	17 (32.7 %)		
University degree	24 (20.0 %)	14 (20.6 %)	10 (19.2 %)		
Age at headache onset (years)	24 (19–26.5)	23.5 (18–26.5)	24 (19–26.5)	*t* = 1.46	0.1381
Duration of chronic pain (years)	16.4 ± 4.4	15.9 ± 4.2	17.1 ± 4.6	*t* = 1.55	0.1252
Number of days with pain/month	23.6 ± 4.7	22.9 ± 4.3	24.3 ± 5.1	*t* = 1.18	0.2397
VAS score	77.6 ± 20.8	78.5 ± 19.9	76.7 ± 21.7	*t* = 1.19	0.2659

Continuous variables are expressed as mean ± SD, except for age and age at headache onset (median and interquartile range). For age and age of headache onset variables Kruskal–Wallis test was used and relative Chi square values are reported. For the other continuous variable *t* test was used. Percentages between groups were compared by the Chi square test

**Table 2:** Overused drugs by MOH patients at baseline

	Total (*n* = 120)	R-group (*n* = 68)	NR-group (*n* = 52)	Test statistics	*p* value
Simple analgesics/NSAIDs					
Acetaminophen	27 (22.5 %)	13 (19.1 %)	14 (26.9 %)	$\chi^{2} = 1.03$	0.3103
Diclofenac	32 (26.7 %)	15 (22.1 %)	17 (32.7 %)	$\chi^{2} = 1.70$	0.1918
Ketorolac	11 (9.2 %)	6 (8.8 %)	5 (9.6 %)	$\chi^{2} = 0.02$	0.8816
Naproxen	18 (15.0 %)	10 (14.7 %)	8 (15.4 %)	$\chi^{2} = 0.01$	0.9178
Nimesulide	16 (13.3 %)	9 (13.2 %)	7 (13.5 %)	$\chi^{2} = 0.00$	0.9712
Piroxicam	13 (10.8 %)	6 (8.2 %)	7 (13.5 %)	$\chi^{2} = 0.66$	0.4179
Tramadol	15 (12.5 %)	8 (11.8 %)	7 (13.5 %)	$\chi^{2} = 0.08$	0.7806
Combination analgesics					
Butalbital + propyphenazone + caffeine	21 (17.5 %)	11 (16.2 %)	10 (19.2 %)	$\chi^{2} = 0.19$	0.6626
Indomethacin + prochlorperazine + caffeine	24 (20.0 %)	11 (16.2 %)	13 (25.0 %)	$\chi^{2} = 1.43$	0.2311
Opioid analgesics					
Acetaminophen + codeine	6 (5.0 %)	3 (4.4 %)	3 (5.8 %)	$\chi^{2} = 0.11$	0.7353
Triptans					
Sumatriptan	8 (6.7 %)	5 (7.4 %)	3 (5.8 %)	$\chi^{2} = 0.11$	0.7304
Zolmitriptan	7 (5.8 %)	3 (4.4 %)	4 (7.7 %)	$\chi^{2} = 0.58$	0.4474
Rizatriptan	12 (10.0 %)	6 (8.8 %)	6 (11.5 %)	$\chi^{2} = 0.24$	0.6232
Eletriptan	11 (9.2 %)	6 (8.8 %)	5 (9.6 %)	$\chi^{2} = 0.02$	0.8816
Frovatriptan	9 (7.5 %)	4 (5.9 %)	5 (9.6 %)	$\chi^{2} = 0.59$	0.4417

The sum of the percentages exceeds 100 % because the majority of patients used more than one simple analgesic. Patients with overuse of a combination of acute medications: *n* = 38 (31.7 %)

As shown in Table 1, of the 120 patients included, 68 (56.7 %) were successfully detoxified (R-group). There were no significant differences between R-group patients and NR-group patients in age, sex, level of education, age at headache onset, duration of chronic headache or number of days with headache per month at baseline. Classes and doses of drugs overused did not significantly differ between the R- and NR-groups at baseline.

The drugs used for prophylactic treatment after detoxification by patients in the R- and NR-groups are reported in Table 3. The use of antidepressants was more significantly higher in the R-group (*χ* ^2^ = 4.10, *p* = 0.0427), whereas antiepileptics (*χ* ^2^ = 3.97, *p* = 0.0462) were the drugs more often used by patients in the NR-group. No statistically significant differences were found in other prophylactic treatments and also for combination of prophylactic treatments, between the two groups. Duration of prophylactic treatment ranged from 9 months to 1 year in the majority of patients (90.8 %) who completed the study and, at 1-year follow-up visit, 101/120 patients (51 in the R-group and 50 in the NR-group) were still under prophylactic treatment. At follow-up, 17 patients in the R-group had discontinued prophylactic treatment (tricyclic antidepressants: *n* = 5, antiepileptics: *n* = 4, beta-blockers: *n* = 2, SSRIs: *n* = 2, combination of prophylactic treatment: *n* = 4), whereas only 2 patients in the NR-group discontinued treatment (tricyclic antidepressants: *n* = 1, beta-blockers: *n* = 1).

**Table 3:** Prophylactic treatment after detoxification programme

	Total (*n* = 120)	R-group (*n* = 68)	NR-group (*n* = 52)	Test statistics	*p* value
Tricyclic antidepressants	24 (20.0 %)	18 (26.5 %)	6 (11.5 %)	$\chi^{2} = 4.10$	0.0427
Beta-blockers	7 (5.8 %)	4 (5.9 %)	3 (5.8 %)	$\chi^{2} = 0.00$	0.9791
Antiepileptics	50 (41.7 %)	23 (33.8 %)	27 (54.0 %)	$\chi^{2} = 3.97$	0.0462
SSRIs	5 (4.2 %)	5 (7.3 %)	0 (0 %)	$\chi^{2} = 3.99$	0.0683
Combination of prophylactic treatments	29 (24.2 %)	14 (20.6 %)	15 (28.8 %)	$\chi^{2} = 1.09$	0.2950

Two-way analysis of variance was performed for frequency of headache and headache score with Responder/Non-Responder as a between-subjects factor and time (baseline vs. follow-up) as a within-subjects factor. The Responder/Non-Responder × Time interaction effect was significant for both in frequency of headache (*F* = 143.49, *p* < 0.001) and in headache score (*F* = 150.9, *p* < 0.001). Significant results were found in post hoc analysis. Comparison between baseline and 1-year follow-up in the R- and NR-groups showed a statistically significant decrease in frequency of headache in the R-group (23.53 ± 3.50 vs. 11.31 ± 5.26 days with headache/month, *t* = 18.42, *p* < 0.001) while in the NR-group there was no significant difference (24.23 ± 2.99 vs. 24.08 ± 2.79 days with headache/month, *t* = 0.20, *p* = 1.000).

VAS scores measured at 1 year was 32.4 ± 12.9 in the R-group and 69.7 ± 21.7 in the NR-group (*t* = 19.64, *p* < 0.01).

Unfortunately, patients with <50 % decrease in headache frequency (all belonging to NR-group) continued to overuse symptomatic medications.

Both groups had the same percentage of aggregated categories of all mood disorders and all anxiety disorders. A similar percentage distribution was found at baseline for individual disorders, including current major depressive episodes, generalized anxiety disorder, panic disorder with or without agoraphobia and current social phobias (Table 4).

**Table 4:** Psychiatric comorbidity of MOH patients at baseline

	Total group (*n* = 120)	R-group (*n* = 68)	NR-group (*n* = 52)	Test statistics	*p* value
Major depressive episode *n* (%)	15 (12.5 %)	9 (13.2 %)	6 (11.5 %)	$\chi^{2} = 0.08$	0.7806
All mood disorders *n* (%)	75 (62.5 %)	43 (63.2 %)	32 (61.5 %)	$\chi^{2} = 0.04$	0.8491
Generalized anxiety disorder *n* (%)	29 (24.2 %)	16 (23.5 %)	13 (25.0 %)	$\chi^{2} = 0.03$	0.8521
Panic disorder *n* (%)	19 (15.8 %)	10 (14.7 %)	9 (17.3 %)	$\chi^{2} = 0.15$	0.6988
Social phobia *n* (%)	27 (22.5 %)	15 (22.1 %)	12 (23.1 %)	$\chi^{2} = 0.02$	0.8947
All anxiety disorders *n* (%)	58 (48.3 %)	33 (48.5 %)	25 (48.1 %)	$\chi^{2} = 0.00$	0.9608

The sum of the percentages exceeds 100 % because some patients had more than one psychiatric disorder

Mean Beck Depression Inventory scores did not differ significantly between R- and NR-groups at the baseline (21.6 ± 11.4 vs. 22.5 ± 10.9, *t* = 1.18, *p* = 0.238) as well as for the Beck Anxiety Inventory scores (22.1 ± 11.3 vs. 23.6 ± 10.8, *t* = 1.18, *p* = 0.238).

At baseline, the mean LDQ total score was slightly higher in the NR-group than in the R-group (12.1 ± 2.1 vs. 11.9 ± 2.0 LDQ total score, *t* = 0.35, *p* = 1.000). Although this difference was not significant at baseline, the LDQ total score was significantly higher in the NR-group than in the R-group at 1-year follow-up (12.1 ± 2.1 vs. 7.8 ± 2.3 LDQ total score, *t* = 0.35, *p* < 0.001). A two-factor MANOVA with all LDQ items as dependent variables has been performed. The overall effects are significant with respect of R/NR-groups (*F* = 15.92, *p* < 0.001), baseline/follow-up (*F* = 7.73, *p* < 0.001), the interaction between R/NR groups and baseline/follow-up (*F* = 7.21, *p* < 0.001).

As shown in Table 5, a statistically significant difference, between the pattern of the responses of the patients in the R-group and that of the NR-group, was found in items 3, 7, 8, 9 and 10. In particular, R-group patients, in items 3 and 9, had significantly lower scores than NR-group patients at both baseline and 1-year follow-up. R-group patients, in item 7, had significantly lower scores than NR-group patients at 1-year follow-up. Furthermore, at 1-year follow-up, R-group patients had significantly lower scores than baseline and NR-group patients in items 8 and 10.

**Table 5:** Leeds Dependence Questionnaire (LDQ) score (mean ± SD) for the R-group and the NR-group at baseline and at 1-year follow-up

Item	R-group		NR-group	
	Baseline	1 year	Baseline	1 year
1. Do you find yourself thinking about when you will next be able to take analgesics?	1.4 ± 0.8	1.0 ± 0.6*	1.3 ± 0.9	1.3 ± 0.9
2. Is taking analgesics more important than anything else you might do during the day?	0.8 ± 0.7	0.7 ± 0.6	1.0 ± 0.7	0.9 ± 0.6
3. Do you feel your need for analgesics is too strong to control?	0.9 ± 0.8†	0.6 ± 0.6‡	1.3 ± 0.7	1.4 ± 0.6
4. Do you plan your days around taking analgesics?	0.9 ± 0.5	0.6 ± 0.5†	1.0 ± 0.7	1.0 ± 0.7
5. Do you take analgesics in a particular way in order to increase the effect it gives you?	0.7 ± 0.7	0.6 ± 0.6	0.7 ± 0.7	0.7 ± 0.7
6. Do you take analgesics morning, afternoon and evening?	1.4 ± 0.5	1.0 ± 0.5**^,^‡	1.5 ± 0.5	1.5 ± 0.5
7. Do you feel you have to continue taking analgesics once you have started?	1.0 ± 0.7	0.8 ± 0.6^‡^	1.3 ± 0.6	1.3 ± 0.6
8. Is getting the effect you want more important than the particular analgesic you use?	2.1 ± 0.6	1.0 ± 0.7**^,^‡	1.8 ± 0.7	1.8 ± 0.7
9. Do you want to take more analgesics when the effect starts to wear off?	0.9 ± 06^‡^	0.7 ± 0.5^‡^	1.6 ± 0.7	1.5 ± 0.7
10. Do you find it difficult to cope with life without analgesics?	1.9 ± 0.7^‡^	1.1 ± 0.6**	1.3 ± 0.7	1.2 ± 0.6
LDQ total score	11.9 ± 2.0	7.8 ± 2.3**^,‡^	12.8 ± 2.1	12.1 ± 2.1

Differences within each group at baseline and at 1-year follow-up: * *p* ≤ 0.01, ** *p* < 0.001

Differences between the R- and NR-groups at baseline and at 1-year follow-up: ^†^ *p* ≤ 0.007, ^‡^ *p* < 0.001

## Discussion

In the present study, we used the LDQ to assess cognitive and behavioural features of substance dependence in a group of MOH patients attending our headache centre. A different pattern of responses was observed between MOH patients who continued to overuse symptomatic medications and therefore retained a chronic pattern of headache (NR-group) and those patients who showed an improvement ≥50 % in headache days/month from baseline, not overusing symptomatic drugs and without any relapse at 1-year follow-up (R-group).

Although the LDQ total score at baseline was not statistically different between the R- and the NR-groups, specific items were statistically significant. The NR-group had the highest subscores for items 3 and 9, which measure, respectively, the inability to refrain from using a substance and the need to continue its administration to maintain well-being, therefore fulfilling DSM-IV criteria [25] for dependency. Indeed, item 3 investigates the *compulsion to start* to take drugs, which concerns a persistent desire or failure to cut-down on substance use, whereas item 9 measures the *constancy of state*, investigating the need to maintain a constant drug effect.

At 1-year follow-up, the LDQ total score was statistically lower in the R-group than in the NR-group, which means that this type of psychometric testing can carefully assess the pathological substance use as well as the treatment efficacy. Specifically, the R-group, compared with the NR-group at 1-year follow-up, no longer showed the need to organize their day around obtaining and using the substance, or continue using the substance in order to enhance or prolong the state achieved by initial use, as shown by statistically lower subscores for items 4, 6, 7 and 8. Particularly, item 8 investigates the *primacy of effect*, indicating that any pharmacological effect obtained by the used drug was more important than the resolution of a specific problem.

Furthermore, the R-group showed a statistically higher subscore at baseline compared with the NR-group for item 10. This subscore drastically fell at 1-year follow-up in R-group but did not significantly vary in NR-group at 1 year. Item 10 assesses the *cognitive set*, 1concerning that drug use is needed to cope with everyday life and without its intake the person’s existence might not be possible.

Therefore we can postulate that, patients in R-group consider the abused drugs to overcome chronic head pain and necessary to return to a normal functioning. This might indicate a different perception of the overused painkillers among the two groups: in the responder one the perception changed to positive when an improvement of headache is achieved with an adequate headache management; otherwise, it is not modified in Non-Responder patients.

Therefore, in our study, patients with unsuccessful outcome (NR-group) had, in some items, a LDQ subscore similar to that found in addicts in items assessing compulsive dependence according to DSM-IV criteria [18, 25, 29].

Conversely, in R-group patients, drug-dependence behaviour seemed to be more a consequence of the chronic headache, reflecting the need for daily analgesic use to cope with everyday life; their LDQ scores at 1-year follow-up were similar to those found in episodic migraine in a previous study [21].

Based on the above results, we believe that there are two different subgroups in MOH patients: those with a substance *abuse* as the persistence of adverse social, psychological or medical consequences related to the repeated use of substances and who were responders to treatment, and those with substance *dependence*, identified by physiological markers of withdrawal and tolerance effects and who were Non-Responders to the treatment, leading to the resumption of medication and seemed to have a dependence behaviour similar to that of a drug addict.

In these NR-group patients, mechanisms underlying sensitization could be more similar to those described for other forms of drug addiction [18].

Moreover, cognitive impulsivity in drug overuse patients seems more strictly associated with dysfunction of the fronto-striatal system resembling the compulsive reward-seeking of addicts [30]. These peculiar aspects could explain the difficulty in avoiding overuse substances and the low compliance of these patients.

The different prophylactic treatments used by MOH patients might have some influence on the results. In fact, in the R-group the use of antidepressant drugs was higher than in the NR-group. Interestingly, most of the antidepressant drugs influence the striato-thalamo-orbitofrontal circuit [31], that is also involved in the pathogenesis of MOH and drug-seeking behaviour [30, 32–37].

Behavioural correlates of some MOH patients might in part resemble some of the characteristics of the behavioural sensitisation to psycho-stimulants [16, 18, 38]. Among these characteristics, the most important are the need to repetitively take drugs during a certain period of time and the occurrence of cross-sensitisation among different drugs used to treat headache. In addition, in these subjects, the possibility of a relapse after relatively long periods of abstinence suggests a vulnerability to drug dependence [39, 40]. This seems to be the prevailing mechanism underlying the inefficacy of the detoxification regimen and prophylactic treatment in the NR-group.

The distinguishing factor of the present study from past studies is the use of the LDQ to better characterize the patients with MOH and to find some dependence traits which have an impact on treatment regimen. For this reason, it is our opinion that MOH patients need an integrated treatment programme that should aim to address both substance *abuse* and *dependence* in parallel. This recommendation necessitates the accurate clinical assessment of substance *dependence* in this population.

In particular, the LDQ subscores can be useful to verify the pattern of dependence in patients with MOH which could be different between patients satisfactorily responding to the treatment and those who do not.

This study has some limits which need to be addressed: LDQ has not yet been validated for the analgesics dependence and thus further validity studies are required. Sensitivity and specificity of each LDQ items in predicting the outcome of the MOH patients should be investigated; furthermore, patients were evaluated neither for other risk factors for relapse to drug overuse nor for other comorbidities, especially for other chronic painful disorders overusing analgesics (such as fibromyalgia, low back pain or neuropathic pain). These aspects should be fully clarified in future research.
